# Evolution of Cerebral Atrophy in a Patient with Super Refractory Status Epilepticus Treated with Barbiturate Coma

**DOI:** 10.1155/2017/9131579

**Published:** 2017-01-15

**Authors:** Christopher R. Newey, Pravin George, Premkumar Nattanmai, Christine Ahrens, Stephen Hantus, Aarti Sarwal

**Affiliations:** ^1^Department of Neurology, University of Missouri, 5 Hospital Drive, CE 540, Columbia, MO 65211, USA; ^2^Cleveland Clinic, Department of Neurology, Cerebrovascular Center, 9500 Euclid Avenue, Cleveland, OH 44125, USA; ^3^Cleveland Clinic, Department of Pharmacy, 9500 Euclid Avenue, Cleveland, OH 44125, USA; ^4^Cleveland Clinic, Department of Neurology, Epilepsy Center, 9500 Euclid Avenue, Cleveland, OH 44125, USA; ^5^Wake Forest University School of Medicine, Neurology and Critical Care (Anesthesia), Reynolds M, Medical Center Blvd, Winston Salem, NC 27157, USA

## Abstract

*Introduction*. Status epilepticus is associated with neuronal breakdown. Radiological sequelae of status epilepticus include diffusion weighted abnormalities and T2/FLAIR cortical hyperintensities corresponding to the epileptogenic cortex. However, progressive generalized cerebral atrophy from status epilepticus is underrecognized and may be related to neuronal death. We present here a case of diffuse cerebral atrophy that developed during the course of super refractory status epilepticus management despite prolonged barbiturate coma.* Methods*. Case report and review of the literature.* Case*. A 19-year-old male with a prior history of epilepsy presented with focal clonic seizures. His seizures were refractory to multiple anticonvulsants and eventually required pentobarbital coma for 62 days and midazolam coma for 33 days. Serial brain magnetic resonance imaging (MRI) showed development of cerebral atrophy at 31 days after admission to our facility and progression of the atrophy at 136 days after admission.* Conclusion*. This case highlights the development and progression of generalized cerebral atrophy in super refractory status epilepticus. The cerebral atrophy was noticeable at 31 days after admission at our facility which emphasizes the urgency of definitive treatment in patients who present with super refractory status epilepticus. Further research into direct effects of therapeutic coma is warranted.

## 1. Introduction

Status epilepticus is a neurological emergency with significant morbidity and mortality [1]. It can be refractory to standard first- and second-line medications in 23–43% cases [2]. If status epilepticus continues after the induction of anesthesia, it is termed super refractory status epilepticus [3]. Super refractory status epilepticus itself portends many long-term complications and sequelae now increasingly being recognized [3, 4].

Focal cerebral atrophy is a well-known phenomenon after prolonged seizures or status epilepticus. However, global cerebral atrophy is less characterized. Global cerebral atrophy in refractory status epilepticus may be a result of prolonged critical illness, progression of underlying brain pathology causing seizures independent of the status epilepticus, or direct effect of high doses of antiepileptic agents used to treat the status epilepticus [4, 5]. Elucidation of pathophysiology and triggers might help in emphasizing clinical paradigms of management that lead to better outcomes. We present a case of progressive generalized cerebral atrophy in a patient with super refractory status epilepticus treated with prolonged barbiturate coma.

## 2. Case

**Figure 1:**
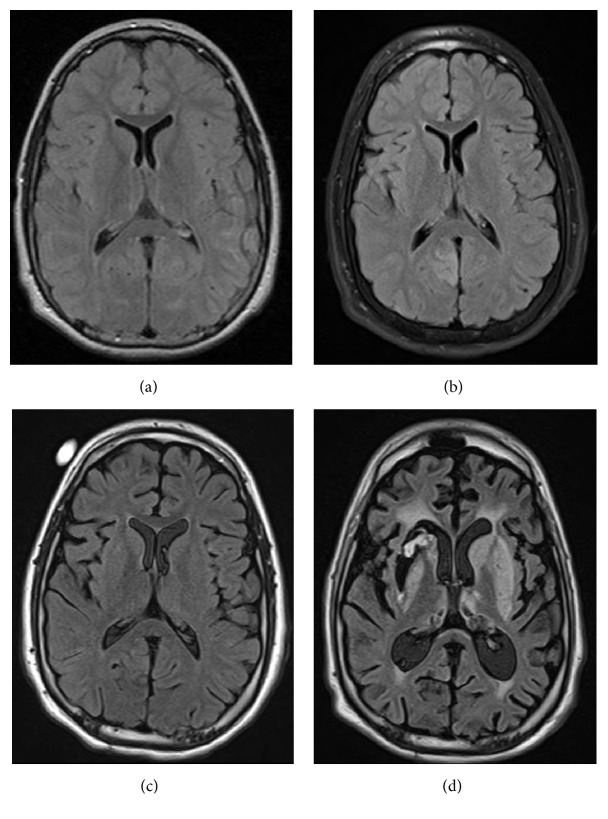
Serial brain magnetic resonance imaging (MRI). Consecutive fluid attenuated inversion recovery sequences (FLAIR) of brain MRIs of the patient with super refractory status epilepticus in a medically induced coma. The serial images show development and progression of diffuse cerebral atrophy.

The patient is a 19-year-old male with a past medical history of autism (reads at 3rd/4th grade level), lower extremity spasticity, and unclassified epilepsy syndrome since early childhood. Prior epilepsy workup (including FISH, SNP array, and MECP2 sequencing) was unremarkable as well as a magnetic resonance imaging (MRI) of the brain 5 years prior to current admission (Figure 1(a)).

He presented to an outside facility initially with altered mental status, suggestive of a stroke-syndrome, along with focal clonic seizures of his right face along with nystagmus. While at the OSH, he required sequential addition of benzodiazepine, phenytoin, valproic acid, and levetiracetam for his seizures. His seizures were refractory to these therapies, and propofol infusion was started. He was eventually transferred to our hospital.

Ongoing nonconvulsive seizures originating from the left hemisphere were seen on continuous electroencephalography (EEG). Due to failure of multiple agents, including propofol, therapeutic barbiturate coma was initiated on the 11th day from status epilepticus onset. He was maintained in a pentobarbital-induced coma for 62 days with multiple failed attempts at weaning. The maximum rate of the pentobarbital infusion was 7.5 mg/kg/hr. The pentobarbital was weaned systematically with first attempt at 24 hours from initiation and then 3 days and 7 days from start of barbiturate coma. Further attempts were then made during this time as clinically allowed as anticonvulsants were introduced and optimized. Additional anticonvulsants included lacosamide, topiramate, ketogenic diet, phenobarbital (level 51.5 mcg/mL), oxcarbazepine, and felbamate. After 62 days on pentobarbital, he developed septic shock requiring vasopressor support and broad spectrum antibiotics. He was weaned off the pentobarbital and started on midazolam infusion. He remained on midazolam infusion for additional 33 days.

His hospital course was further complicated by critical illness myopathy and neuropathy, angioedema, anasarca, exposure keratitis, prolonged mechanical ventilation (82 days on ventilator), ventilator associated pneumonia and urinary tract infection (both requiring antibiotic therapy), critical illness related adrenal insufficiency, prolonged hypothermia, ileus, transaminitis, lactic acidosis (propylene glycol level was 58 mg/dL), acute renal failure requiring continuous venovenous hemodialysis, acute blood loss anemia, and ultimately need for tracheostomy and percutaneous gastrostomy.

Given his past history, there was concern for an undiagnosed metabolic disorder as the etiology for his unclassified epilepsy syndrome. He was started on arginine, carnitine, riboflavin, coenzyme Q10, folinic acid, creatinine, vitamin E, and vitamin C. He had an extensive workup for the etiology of his status epilepticus. Plasma amino acids, acylcarnitine, pyruvate, ammonia, and urine acids were obtained and were unremarkable. Mitochondrial DNA screening panel was negative for disease causing mutation. POLG1 sequencing was unremarkable as well as methylmalonic acid, homocysteine, vitamin B12, and sialic acid levels in the urine. Additionally, testing for fluorescence in situ hybridization (FISH), single nucleotide polymorphism (SNP) array, myotonic dystrophy, Charcot-Marie-Tooth, paraneoplastic panel, and thyroid antibodies were unremarkable. Muscle biopsy showed myopathic changes consistent with his critical illness. Lumbar puncture was unremarkable with 0 WBC, 0 RBC, glucose of 53 mg/100 cc, and protein of 21 mg/dL with negative universal PCR and CSF neurotransmitters, amino acids, and sialic acid levels.

Over the course of hospitalization, he had serial MRI brains performed, including an admission MRI at our institution (11 days from onset; Figure 1(b)). Diffuse generalized atrophy was noted in comparison to prior MRI (Figure 1(a)). The diffuse cerebral atrophy significantly progressed by 31 days and 136 days from onset of status epilepticus (Figures 1(c) and 1(d), resp.).

He eventually was discharged to a long-term acute care facility. On neurological examination prior to discharge, he would spontaneously open his eyes but would not follow commands or track examiner. He would not attempt to speak. He had flaccid quadriparesis. He required ventilator support. It remained unclear as to the etiology for his refractory status epilepticus.

## 3. Discussion

This case illustrates the rapid development and progression of global cerebral atrophy in a patient with super refractory status epilepticus requiring pentobarbital coma for a total of 62 days with 11 days of presumed nonconvulsive seizure activity prior to the definitive treatment with pentobarbital. A key finding in this case is the development of cerebral atrophy most notably at 31 days and continued progression of the cerebral atrophy at 136 days.

A recently published study by Hocker et al. correlated the duration of anesthesia for treatment of super refractory status epilepticus with increased ventricular brain ratio (VBR) [4]. The change in VBR appears to be most drastic once the duration of the anesthetic has been nearly 40 days [4]. 68.4% of patients in this study died by 1 year or were lost to follow-up [4]. Only 4 patients had good outcomes defined by modified Rankin score (mRS) of 3 or better. Interestingly, the development of atrophy did not correlate with functional outcome [4]. One patient with N-methyl-D-aspartate receptor encephalitis had “reversible” brain atrophy [4]. Our patient's generalized cerebral atrophy did not reverse. It progressively worsened as seen on the MRI 63 days (i.e., 136 days after initial presentation of status epilepticus) after the stopping of the pentobarbital coma.

The underlying mechanism for the progression of generalized cerebral atrophy in super refractory status epilepticus is largely unknown. Anesthetics have been proposed to cause disruption of the ascending reticular activating system resulting in decoupling and subsequent excitotoxicity and ultimately apoptosis [6]. Additionally, critical illness, such as sepsis, and the chronic use of anticonvulsants have been shown to cause diffuse cerebral atrophy resulting in long-term cognitive impairment [7, 8]. Cerebral atrophy, however, may be from underlying ongoing nonconvulsive seizure activity not detectable on surface electrodes causing ongoing neuronal death [9, 10].

Recognizing complications, either directly or indirectly related, to barbiturate coma are important and may lead to improved outcomes [5, 11]. Future studies should systematically evaluate the long-term functional outcomes in patients who present with super refractory status epilepticus in relation to the management and associated complications. Multiple complications were observed in our patient during his medically induced coma. These complications may not be directly related to the medications themselves itself, but rather a reflection of the immobile, critically ill state [5].

In conclusion, this case highlights the rapid development and progression of generalized cerebral atrophy in a patient with super refractory status epilepticus. The diffuse cerebral atrophy was noted during treatment of his status epilepticus and continued to progress even after pentobarbital coma was discontinued. This highlights the urgency of aggressive intervention in patients who present with status epilepticus. Further research is warranted to enhance the understanding of pathophysiology of ongoing brain atrophy in super refractory status epilepticus and its management with induced coma. Understanding these changes further could lead to clinical paradigms that improve survival and functional outcomes.
